# Comparative analysis of medical glue and positioning hooks for preoperative localization of pulmonary nodules

**DOI:** 10.3389/fonc.2024.1392213

**Published:** 2024-07-04

**Authors:** Haowen Wang, Min Deng, Dexin Cheng, Rui Feng, Hanbo Liu, Tingyang Hu, Dongdong Liu, Cheng Chen, Peilin Zhu, Jian Shen

**Affiliations:** ^1^ Interventional Radiology Department, Zhejiang Provincial People’s Hospital, Hangzhou, China; ^2^ Thoracic Surgery Department, Zhejiang Provincial People 's Hospital, Hangzhou, China; ^3^ Zhejiang Provincial People’s Hospital, People’s Hospital of Hangzhou Medical College, Hangzhou, China

**Keywords:** positioning hooks, medical glue, pulmonary nodules, preoperative localization, clinical value

## Abstract

**Background:**

Through preoperative localization, surgeons can easily locate ground glass nodules (GGNs) and effectively control the extent of resection. Therefore, it is necessary to choose an appropriate puncture positioning method. The purpose of this study was to evaluate the effectiveness and safety of medical glue and positioning hooks in the preoperative positioning of GGNs and to provide a reference for clinical selection.

**Methods:**

From March 30, 2020 to June 13, 2022, a total of 859 patients with a CT diagnosis of GGNs requiring surgical resection were included in our study at the hospital. Among them, 21 patients who either opted out or could not undergo preoperative localization for various reasons were excluded. Additionally, 475 patients who underwent preoperative localization using medical glue and 363 patients who underwent preoperative localization through positioning hooks were also excluded. We conducted statistical analyses on the baseline data, success rates, complications, and pathological results of the remaining patients. The success rates, complication rates, and pathological results were compared between the two groups—those who received medical glue localization and those who received positioning hook localization.

**Results:**

There was no statistically significant difference between the two groups of patients in terms of age, body mass index, smoking history, location of the nodule, distance of the nodule from the pleura, or postoperative pathological results (*P* > 0.05). The success rate of medical glue and positioning hooks was 100%. The complication rates of medical glue and positioning hooks during single nodule positioning were 39.18% and 23.18%, respectively, which were significantly different (*p* < 0.001); the complication rates during multiple nodule positioning were 49.15% and 49.18%, respectively, with no statistically significant differences (*p* > 0.05). In addition, the method of positioning and the clinical characteristics of the patients were not found to be independent risk factors for the occurrence of complications. The detection rate of pulmonary nodules also showed some positive correlation with the spread of COVID-19 during the 2020–2022 period when COVID-19 was prevalent.

**Conclusion:**

When positioning a single node, the safety of positioning hooks is greater than when positioning multiple nodes, the safety of medical glue and positioning hooks is comparable, and the appropriate positioning method should be chosen according to the individual situation of the patient.

## 1 Introduction

Lung cancer is one of the malignant tumors with the highest morbidity and mortality rates worldwide (1). According to global cancer epidemic statistics (GLOBOCAN), there were approximately 2.207 million new lung cancer cases and approximately 1.796 million new lung cancer deaths in 2020, accounting for 11.4% and 18.0% of all new malignant tumor cases and deaths, respectively (2). In recent years, with the increasing health needs of people and the wide application of high-resolution multislice computed tomography (CT) technology (3), the detection of ground glass nodules (GGNs) has become easier. Many of these cases are early lung cancer or precancerous lesions, of which the possibility of malignant tumors is as high as 59%–73% (4). Video-assisted thoracic surgery (VATS) is a new minimally invasive surgical technique that uses endoscopic vision to perform small wound operations (5). It has significant advantages in treating early lung cancer, especially GGN, and has been widely used in clinical practice (6). However, because some GGNs are too small or more than 5 mm from the pleura, they cannot be accurately positioned by finger touch or endoscopic instruments, resulting in a prolonged operation time and increased risk of thoracotomy (7, 8). Therefore, preoperative GGN localization is related to the ability to accurately locate and quickly resect the lesion, which is the key to the success of VATS lung nodule surgery (9).

Various techniques have been used for the effective control of the extent of resected ground glass opacities during VATS, including CT-guided placement of positioning devices (hookwires, microcoils, etc.), percutaneous injection of reagents (methylene blue, medical glue, indocyanine green, etc.), intraoperative ultrasound, and electromagnetic navigation bronchoscopy (ENB) (10). Among the abovementioned positioning methods, medical glue and positioning hooks are the two most commonly used. However, most previous studies have focused on a single method or small-sample analysis, and few large-sample-data analyses have compared the effectiveness, safety, and feasibility of the abovementioned two methods in the localization of single nodules and multiple nodules. Yang Yu et al. compared the advantages and disadvantages of two preoperative localization methods, the pulmonary nodule localization hook (P-N-L-N) and microcoils, in 150 patients treated with VATS. The results showed that the use of P-N-L-N was more effective than the use of microcoils in reducing operative time, intraoperative bleeding, total postoperative drainage, and postoperative discharge time (11). In addition, in a study by Zhang et al. comparing the efficacy and safety of hookwires and medical glue, it was demonstrated that preoperative positioning with medical glue was associated with a lower complication rate, a greater rate of successful positioning, less post-positioning pain, and a flexible surgical schedule (12). The purpose of this study was to analyze the effectiveness and safety of two positioning methods, medical glue and positioning hooks, and to compare the safety and accuracy of these two methods between single nodules and multiple nodules to provide a reference for different types of patients to choose the appropriate method for the preoperative positioning of VATS.

## 2 Methods

### 2.1 Clinical data

**Figure 1:**
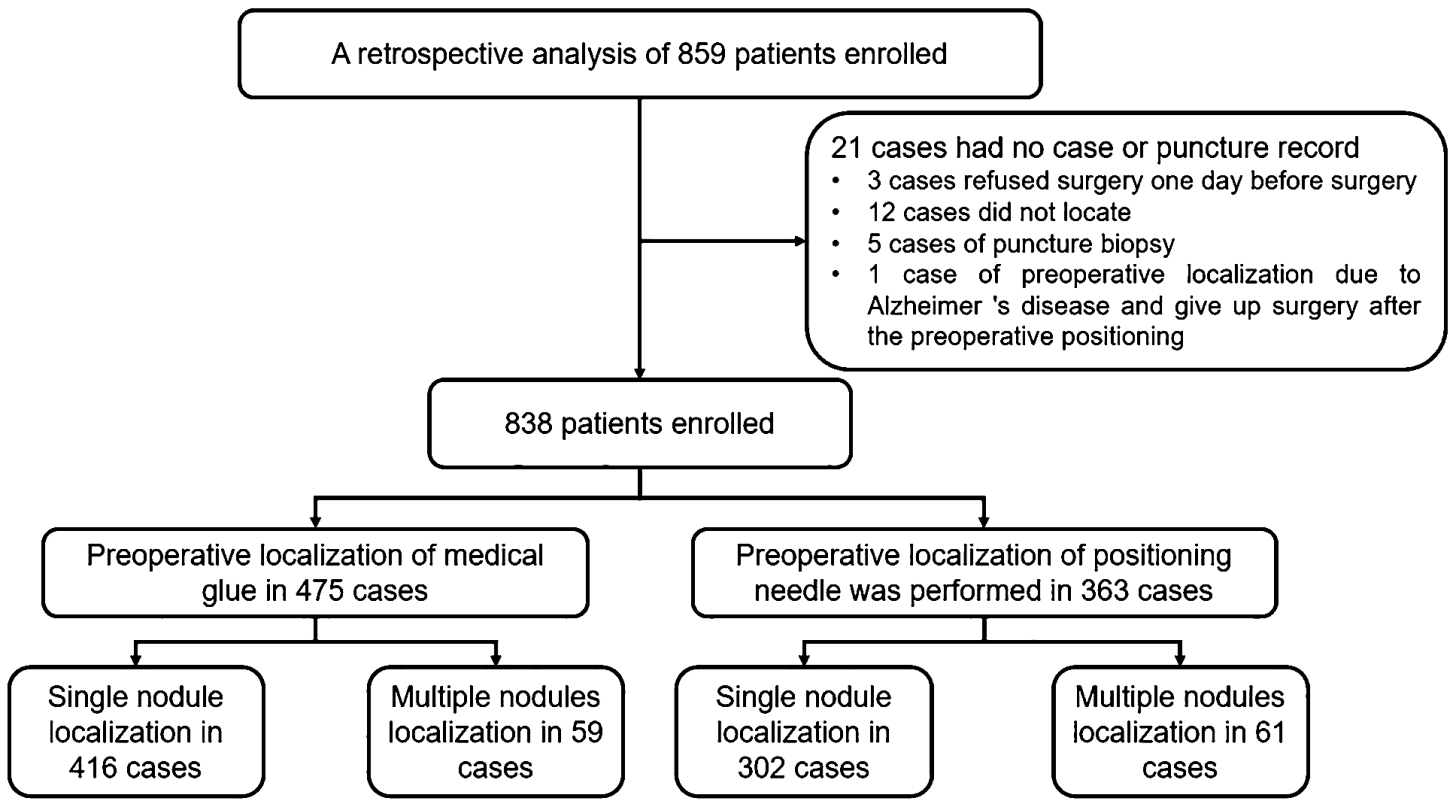
Research process.

This study retrospectively analyzed a total of 859 patients who underwent VATS at our hospital from March 30, 2020 to June 13, 2021, and the enrollment process is shown in **Figure 1**. A total of 21 patients were excluded (three patients refused surgery 1 day before surgery, 12 patients did not undergo surgery, five patients underwent puncture biopsy, and one patient underwent preoperative localization due to Alzheimer’s disease and refused surgery after preoperative positioning). Among the remaining 838 patients, 475 patients underwent preoperative medical glue positioning, including 138 male and 337 female patients. There were 363 patients who underwent positioning hooks, including 100 male and 263 female. There were 416 patients with single node localization by medical glue, 59 patients with multiple nodes (number of nodules ≥2), 302 patients with single node localization by positioning hooks, and 61 patients with multiple nodes. The detailed baseline characteristics of the patients are shown in **Table 1**. The inclusion criteria for the two groups were as follows: (1) a CT diagnosis of ground glass nodules prone to adenocarcinoma *in situ* (AIS), minimally invasive adenocarcinoma (MIA), and invasive adenocarcinoma (IA) was made; (2) no pleural indentation, which is defined as an obvious linear indentation or linear shadow on a lung tumor on CT; and (3) no contraindications to surgery were found on routine preoperative VATS examination. The exclusion criteria were as follows: (1) patients with previous tuberculous pleurisy or imaging findings of extensive pleural adhesions who were unable to undergo VATS and (2) surgical contraindications. The nodule is located in one-third of the lung field or near the hippocampus blood vessel or bronchus, and the visual field is blurred.

**Table 1:** Clinical characteristics of the two groups of patients.

	Medical glue group (*N* = 475)	Positioning hooks group (*N* = 363)	*P*-value
**Gender**			0.337
Male	138	100	
Female	337	263	
**Age, years**	52 ± 13	54 ± 13	0.496
**Tumor history**			0.616
Yes	54	22	
No	421	341	
**BMI, kg/m^2^**	23.3 ± 3.0	23.0 ± 3.1	0.644
**Smoking**			0.129
Yes	420	308	
No	55	55	
**Multiple nodule localization**			0.093
Yes	59	61	
No	416	302	
**Size of nodules**	8.9 ± 3.6	9.6 ± 4.0	0.183
**Distance between the nodules and the visceral pleura**	9.2 ± 8.2	10.1 ± 9.0	0.055
Position of nodules			
LUL	146	127	
LLL	82	73	
RUL	173	124	
RML	99	84	
RLL	25	24	

LUL, left upper lung, LLL, left lower lung; RUL, right upper lung; RML, right middle lung; RLL, right lower lung.

### 2.2 Medical glue positioning

**Figure 2:**
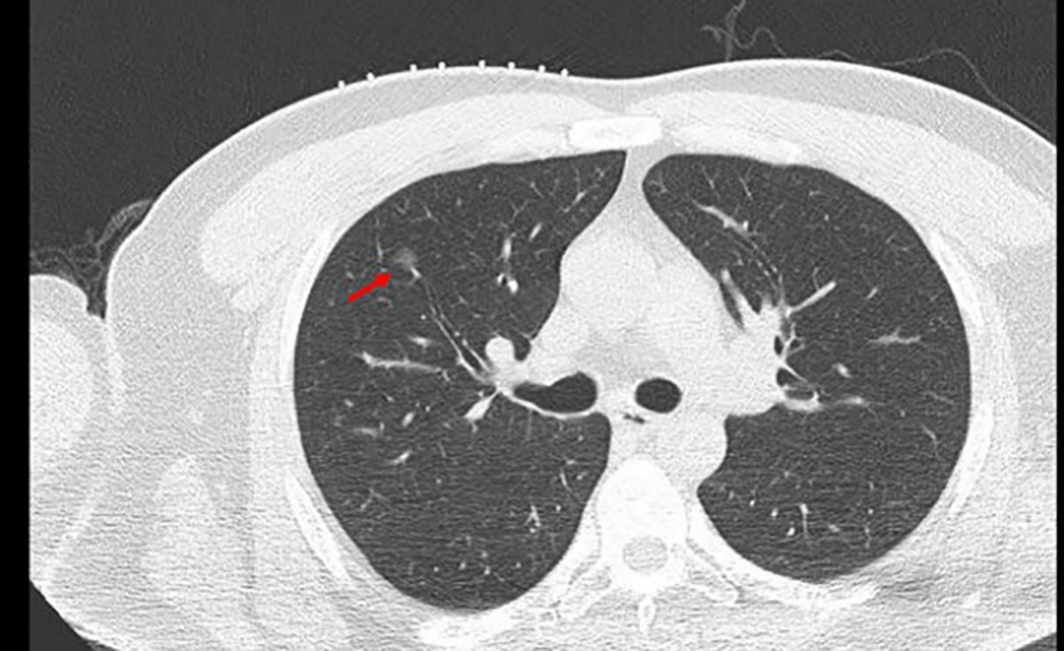
A ground glass nodule with small diameter, low density, and far away from the edge of the pleura. →, location of GGN; GGN, ground glass nodules.

The patient underwent routine chest CT scanning before surgery (**Figure 2**). The relationships between the lesion and the blood vessels and bronchi were observed, and the appropriate scanning parameters and positions were determined. The surgeon performed CT-guided percutaneous puncture injection of medical glue for positioning and marking.

**Figure 3:**
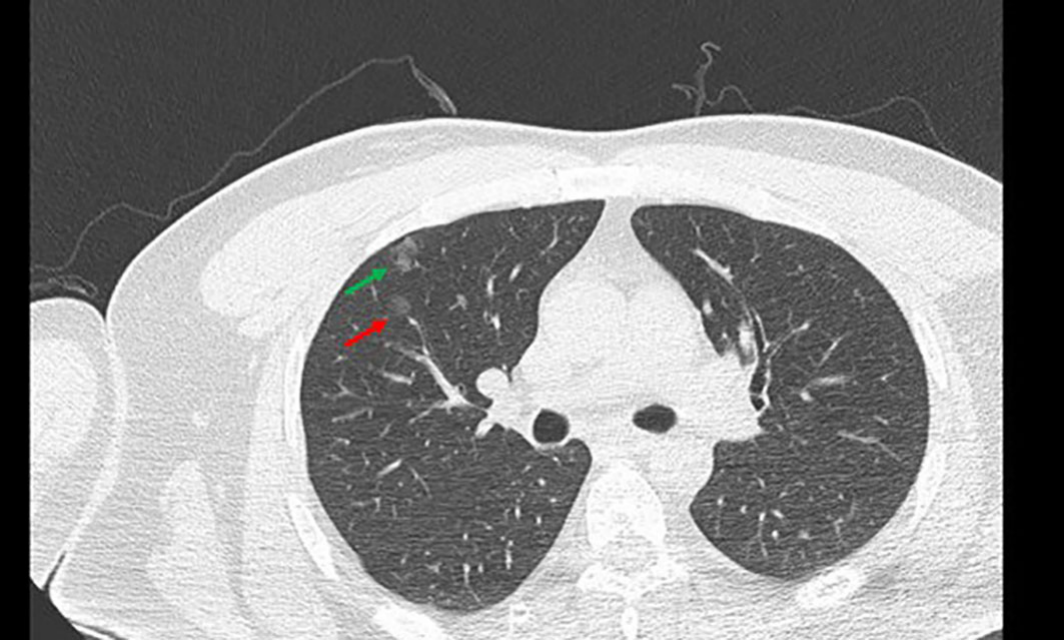
Preoperative positioning with medical glue. the red →, the location of GGN; the green →, medical glue; GGN, ground glass nodules.

The specific operation involved exercising the patient’s respiratory condition before the operation. Before puncture, a Philips CT scan was obtained with the assistance of a fence to determine the puncture point and mark it. Disinfect the puncture point and its surroundings with 75% alcohol with the puncture point as the center, followed by wiping it clean with disinfectant paper towels remaining alcohol for the puncture operation. A 2-mL syringe was used to extract 2% lidocaine, and the pleura was anesthetized layer by layer at the puncture location. The syringe needle was left on the patient, and the CT was rescanned. The scanning range was 3 cm up and down with the puncture layer at the center, and the layer thickness was 2 mm to reduce the radiation on the patient while scanning the nodules as much as possible. The position between the syringe needle and the nodule was observed after scanning. The angle was adjusted, and the depth was calculated. At the same time, the location of the formation was marked after the injection of medical glue, usually 5 mm outside the lung field of the lesion. With an 18-G Chiba puncture needle (GALLINI S.R.L.), the needle was inserted in the direction of the original 2-mL syringe needle. The CT scanner was rescanned to determine the arrival of the expected marking point, the core of the Chiba puncture needle was pulled out, and the medical glue (Beijing Compont Medical Devices Co., Ltd.) was injected. The injection speed was 0.15–0.2 mL with a 1-mL syringe, and 0.1 mL of 5% glucose solution was injected to ensure that the residual medical glue inside the Chiba needle was fully injected into the lungs. The needle was left in place for 3–5 s, after which the needle was quickly removed. The puncture point was wrapped with a dressing. Chest CT (**Figure 3**) revealed the position of the medical glue in relation to the nodule and whether there was any complication in the lung. After confirming the patient’s condition, the patient was sent to the operating room for VATS surgical treatment.

### 2.3 Positioning hooks

**Figure 4:**
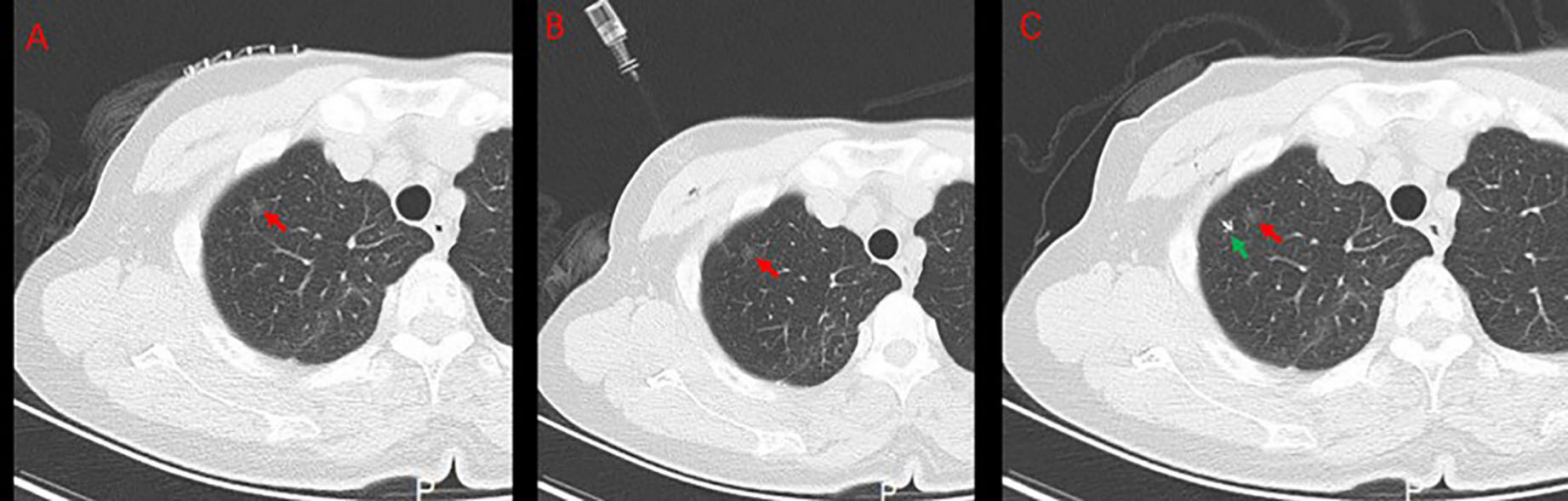
Preoperative positioning with a positioning hook. **(A)** Fence skin localization. **(B)** Positioning needle insertion. **(C)** Positioning hook and GGN after releasing the positioning hook. the red → , the location of GGN; the green → , positioning hooks; GGN, ground glass nodules.

The patient underwent routine chest CT scanning before the operation, the relationship between the lesion and blood vessels and bronchial tubes was observed, the appropriate scanning parameters and position were used, and the operator placed the localization hook under CT-guided percutaneous puncture. Specifically, the patient’s respiratory condition was evaluated before the operation. Before puncture, a Philips CT scanner was used with the assistance of a fence, the puncture point was determined and marked, and the towel was disinfected and spread with the puncture point at the center. A 2-mL syringe was used to extract 2% lidocaine, and the pleura was anesthetized layer by layer at the puncture location. The syringe needle was left on the patient, and CT was rescanned. The scanning range was 3 cm above and below the center of the puncture layer, and the thickness of the layer was 2 mm to reduce the radiation on the patient while simultaneously scanning the nodule. After scanning, the positional relationship between the syringe needle and the nodule was observed. The angle was adjusted, and the depth was calculated. At the same time, the position of the localization hook and the nodule after placement was predicted, and the position was usually selected to be 5 mm outside the lung field of the lesion. Then, chest CT was performed (**Figure 4**), the angle was adjusted, and an 18-G localization needle (Senscure Pulmonary Nodules Localization Needle SS510–10) was used to puncture the edge of the lung nodule. CT was used to confirm that the localization needle reached the predetermined position, after which the localization hook was released and a bandage was applied. Finally, another CT scan was performed to observe the relationship between the localization hook and the nodule and the complications. The patient was sent to the operating room for VATS.

### 2.4 Observation indicators

(1) The surgical location, complications, and positioning success rate were recorded. The complications included pneumothorax, bleeding, cough, indistinguishable bleeding from glue, leakage of glue into the pleura, and pain. Hemoptysis occurred in a very small number of patients. Pneumothorax: Patients who presented with varying degrees of lung compression (defined as the presence of at least ≥10 mm of lung compression) in the lung tissue after CT-guided medical glue or positioning hooks. Bleeding: Patients who presented with a cloudy sign (≥10 mm in maximum diameter) at the puncture site on imaging after CT-guided medical glue or positioning hooks puncture localization. The bleeding of medical glue is a cloudy sign that appears before the injection of medical glue after the puncture needle is in place. Cough: Patients with cough after preoperative positioning; indistinguishable bleeding from glue. Bleeding appeared before injecting medical glue after the puncture needle was put in place during CT-guided medical glue puncture localization, and after the injection of medical glue, the scope of the cloudy sign increased; the overlap of the medical glue and the original bleeding site made it indistinguishable. Leakage of glue into the pleura: After the patient was injected with medical glue under the guidance of CT, the imaging showed a white sign on (≤1 mm) the part of the contact between the puncture needle and the pleura. Pain: Scoring was performed using the Number Rating Scale (NRS), with the numbers from 0 to 10 indicating the pain from no pain to the most. In this study, the complications of pain were recorded only for patients with a pain score ≥4. Nodule coverage: Imaging findings showed that medical glue or positioning hooks coincided with nodules.

(2) The postoperative pathological results were recorded. The description was based on the pathological diagnosis, which was mainly divided into benign and malignant lesions. The benign lesions included inflammatory lesions, atypical adenomatous hyperplasia, hamartoma, sclerosing hemangiomas, fibroplasia, lymphadenopathy, granulomatous inflammation, and alveolar epithelial hyperplasia, and the malignant lesions included bronchial adenoma, adenocarcinoma infiltrating, minimally invasive adenocarcinoma, adenocarcinoma *in situ*, squamous cell carcinoma, and metastatic carcinoma.

### 2.5 Statistical analysis

The descriptive statistics for continuous variables are expressed as mean ± SD. Continuous variables were analyzed using *t*-tests, and categorical variables were compared using chi-square tests or Fisher’s exact tests. A *P*-value less than 0.05 was considered to indicate statistical significance. Multivariate logistic regression analysis was used to analyze the independent risk factors for complications, and *P* < 0.05 was considered to indicate statistical significance. SPSS 26.0 was used to analyze the data.

## 3 Results

### 3.1 Clinical characteristics

A total of 838 patients who underwent preoperative localization were included in the analysis. The detailed characteristics of the patients are shown in **Table 1**. There were 475 patients in the medical glue group, including 138 male and 337 female, with an average age of 52 ± 13 years (18–85 years) and a body mass index (BMI) of 23.3 ± 3.0 years (14.38–35.42). There were 363 patients in the positioning hooks group, including 100 male and 263 female, with an average age of 54 ± 13 years (20–84 years) and a BMI of 23.0 ± 3.1 (17.12–34.24). Among the patients in the medical glue group, 48 had a history of cancer (refer to **Table 1** for further details). Moreover, 55 patients had a history of smoking; among the patients in the positioning hooks group, 21 had a past history of tumors (refer to **Table 1** for further details), and 55 had a history of smoking. There was no significant difference between the two groups in terms of sex, age, smoking history, or BMI (*P* > 0.05).

### 3.2 Comparison of nodules between the two groups

The nodules of the two groups of patients are shown in **Table 1**. In the medical glue group, there were 416 patients with single nodules positioned and 59 patients with multiple nodules positioned. The average diameter of the lung nodules was 8.9 ± 3.6 mm (range, 2.5–26 mm), and the average distance of the nodules from the pleura was 9.2 ± 8.2 mm (range, 4–31 mm). In the positioning hooks group, 302 patients underwent single nodal localization, 61 patients underwent multiple nodal localization, and the average diameter of the lung nodules was 9.6 ± 4.0 mm (range, 4–31 mm) according to CT. In the positioning hooks group, there were 302 patients with single nodule positioning and 61 patients with multiple nodule positioning. CT revealed that the average diameter of the lung nodules was 9.6 ± 4.0 mm (range, 0–40 mm), and the average distance of the nodules from the pleura was 10.1 ± 9.0 mm (range, 0–39.9 mm). There was no significant difference in nodule diameter, nodule size, or nodule-to-pleural distance between the two groups (*p* > 0.05). In the two groups of patients, the number of left upper nodules and right upper nodules was significantly greater than the number of nodules in other locations, but there was no significant difference between the two groups.

### 3.3 Comparison of the complication rate between the two groups

The success rate of preoperative nodal localization was 100% in both groups. There were 146 patients (35.10%) with postoperative complications among the patients with single nodule localization in the medical glue group and 62 patients (20.53%) with postoperative complications among the patients with single nodule localization in the positioning hooks group (**Table 2**).

**Table 2:** Incidence of complications of single nodule localization between the two groups of patients.

	Medical glue group (*n* = 416)	Positioning hooks group (*n* = 302)	*P*-value
**No**	270	240	0.001
**Yes**	146	62	

**Figure 5:**
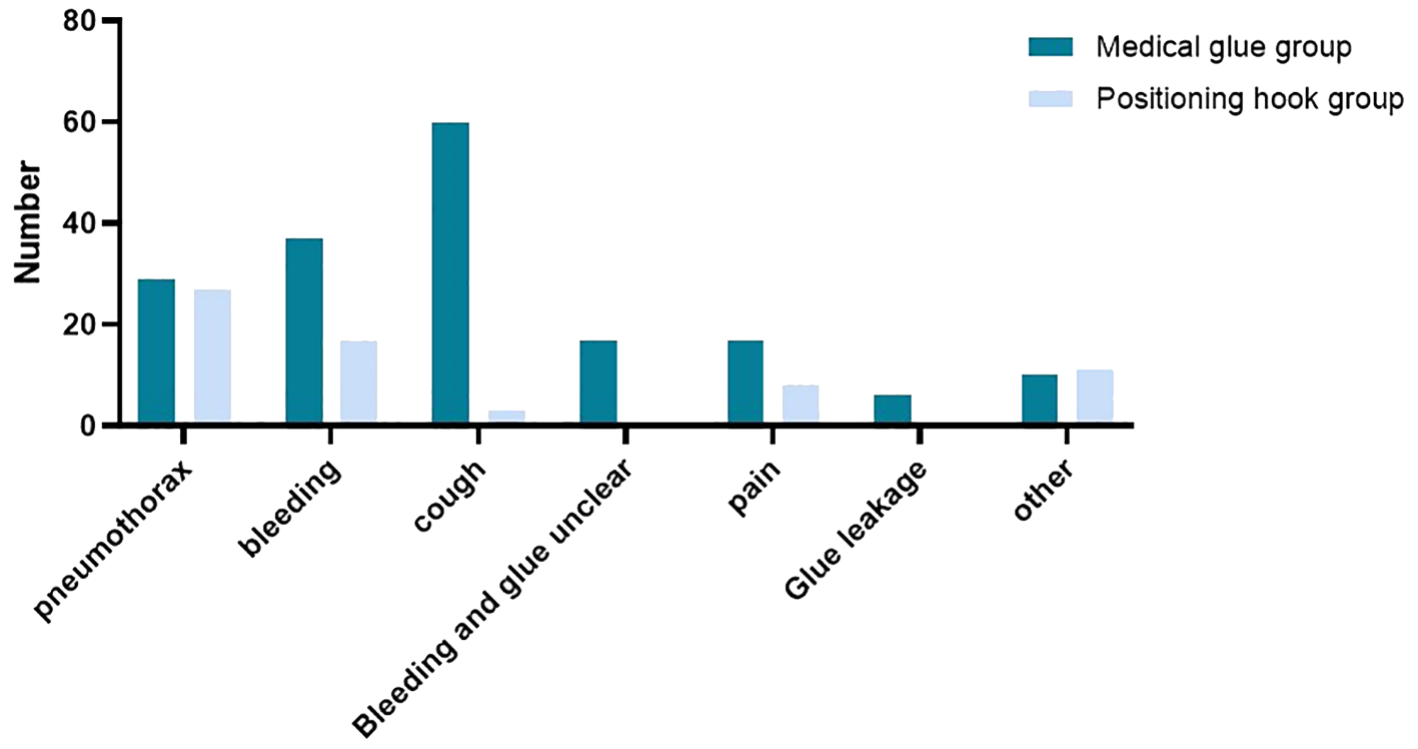
Complications of patients with single nodule localization in the two groups.

There was a significant difference between the two groups (*P* < 0.001). Some patients had multiple complications at the same time, and the detailed statistics are shown in **Figure 5**. Among the patients with complications, 37 had bleeding, 60 had coughing, 17 had bleeding indistinguishable from glue, 17 had pain, six had glue dripping, and 10 had other symptoms (eight cases of nodal coverage) in the medical glue group, whereas in the localization hooks group, 17 had bleeding, three had cough, 11 had pain, and 11 had other symptoms (10 cases of nodal coverage). Among the 29 patients (6.97%) who developed pneumothorax in the medical glue group, the distance between the pleura and the lung was more than 5 cm in four patients, whereas in the positioning hooks group, the distance between the pleura and the lung was more than 5 cm in three of the 27 patients (8.94%) who developed pneumothorax. The study also statistically analyzed the incidence of different types of complications between the two groups. The results showed that there was a significant difference in the occurrence of cough in patients between the two groups (*p* < 0.001), but there was no significant difference in bleeding, pain, or pneumothorax (*p* > 0.05).

For patients with multiple nodules, the probabilities of postoperative complications in the medical glue group and the positioning hooks group were 22 patients (37.29%) and 30 patients (49.18%), respectively (**Table 3**).

**Table 3:** Incidence of complications in patients with multiple nodule localizations in the two groups.

	Medical glue group (*n* = 59)	Positioning hooks group (*n* = 61)	P-value
**No**	37	31	0.799
**Yes**	22	30	

**Figure 6:**
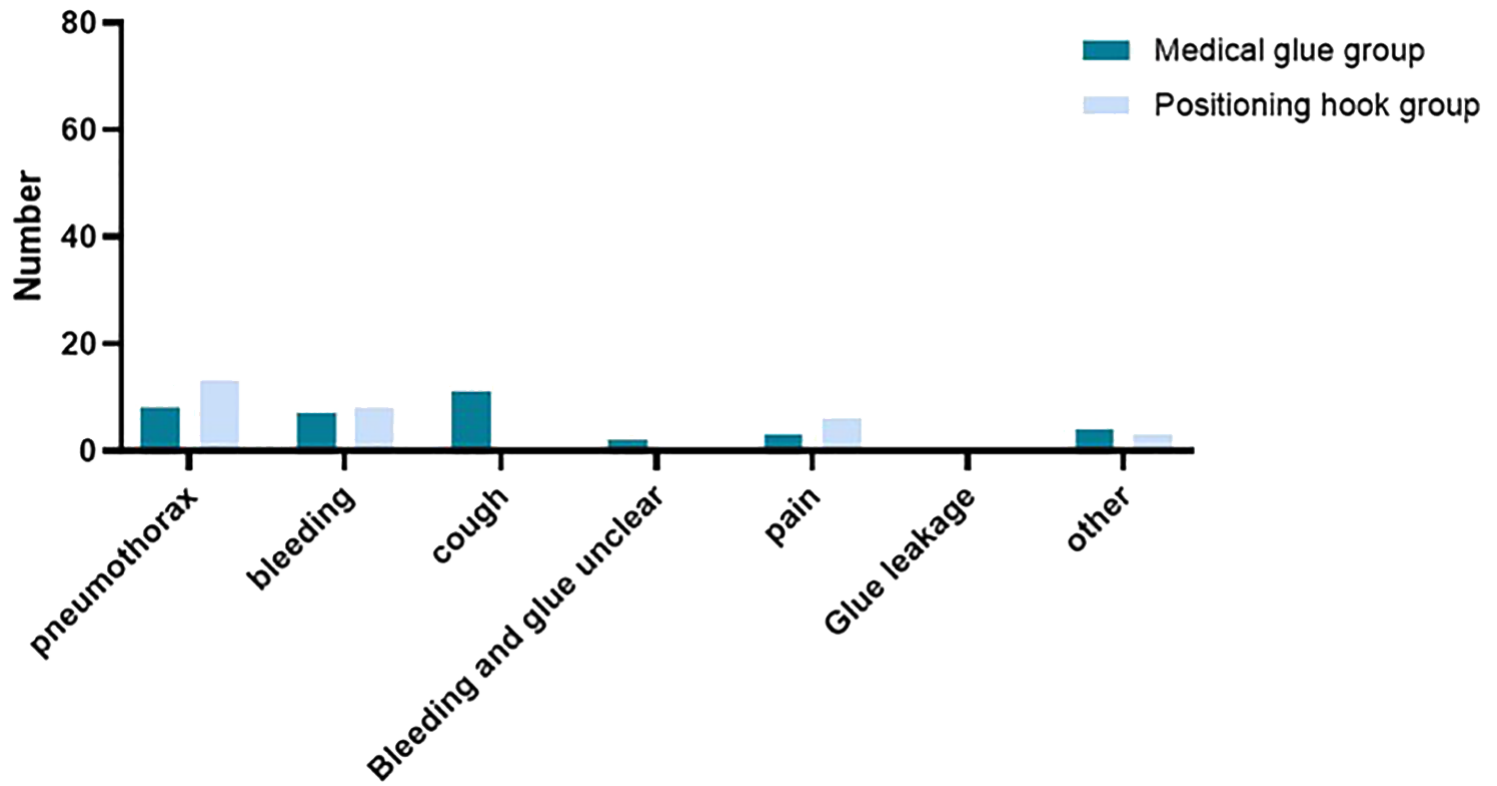
Complications of patients with multiple nodule localization in the two groups.

There was no significant difference between the two groups (*P* = 0.799). Some patients had multiple complications at the same time, and the detailed statistics are shown in **Figure 6**. In the medical glue group, there were eight cases of pneumothorax, seven cases of hemorrhage, 11 cases of coughing, three cases of pain, two cases of indistinguishable bleeding from glue, and four cases of other symptoms (four cases of nodular coverage), and in the positioning hooks group, there were 13 cases of pneumothorax, eight cases of hemorrhage, six cases of pain, and three cases of other symptoms (three cases of nodular coverage). Pneumothorax and hemorrhage were the most common complications in both groups of patients who underwent multiple nodal puncture localization. In the medical glue group, two patients (3.39%) had pneumothorax in which the distance between the pleura and the lung was more than 5 cm, while in the localization hooks group, eight patients (13.11%) had this condition. Similar to the results of the single node complication analysis, there was a significant difference in the incidence of cough between the two groups (*P* = 0.004) but no significant difference in bleeding, pain, or pneumothorax (*P* > 0.05).

These results indicate that the safety of positioning hooks is greater when performing single nodal localization, and there is no significant difference in the safety of the two methods when positioning multiple nodules.

### 3.4 Relationships between the general clinical characteristics of patients and the size, location, and distance of lung nodules from the pleura

The present study analyzed the relationships between patient age, BMI, and smoking status and the size, location, and distance of lung nodules from the pleura. The results are shown in **Table 4**. There was a correlation between patient age, BMI, smoking status, and the size of pulmonary nodules (*p* < 0.05), but there was no significant correlation between the location of pulmonary nodules and the distance from the pleura (*p* > 0.05).

**Table 4:** Correlations between age, body mass index, smoking history, and pulmonary nodules in patients.

Variables		Size of nodules	Distance from pleura
**Age**	*P*-value	0.001	0.281
**Body mass index**	*P*-value	0.005	0.519
**Smoking**	*P*-value	0.001	0.504

### 3.5 Independent risk factors for the occurrence of preoperative localization complications

Multivariate logistic regression was used to analyze whether patient age, sex, nodule size, distance of the nodule from the pleura, positioning method, smoking status, BMI, and location of the pulmonary nodule were independent risk factors for complications. The results of the analysis are shown in **Table 5**. None of the above-mentioned patient characteristics were independent risk factors influencing the occurrence of preoperative positioning complications (*p* > 0.05).

**Table 5:** Multivariate logistic regression analysis of independent risk factors for complications.

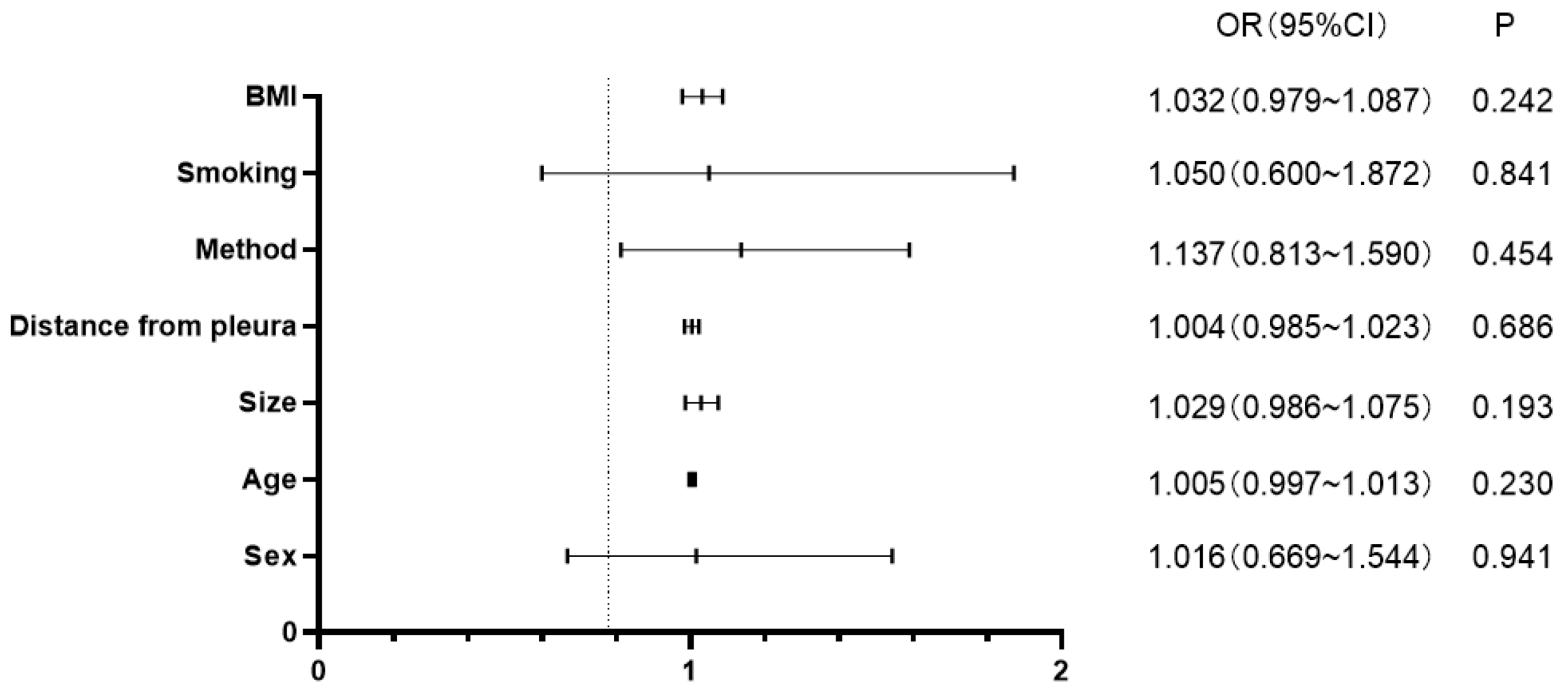

### 3.6 Comparison of pathologic results between the two groups

In patients with a single nodule, the proportions of benign lesions in the medical glue group and localization hooks group were 19.71% and 16.56%, respectively, and the proportions of malignant lesions in the two groups were 80.29% and 83.44%, respectively (**Table 6**). Among the patients with multiple nodules, the percentages of patients with benign lesions in the medical glue group and the positioning hooks group were 22.45% and 16.13%, respectively; the percentages of patients with malignant lesions in the two groups were 77.55% and 83.87%, respectively (**Table 7**). Among them, benign lesions were dominated by atypical adenomatous hyperplasia, fibrous tissue hyperplasia, and alveolar epithelial hyperplasia, while malignant lesions were dominated by microinvasive adenocarcinoma, adenocarcinoma *in situ*, and invasive adenocarcinoma, and the proportion of malignant lesions was significantly greater than that of benign lesions (the proportion of malignant lesions to total lesions reached more than 75%). The specific pathologic results are shown in **Tables 6**, **7**.

**Table 6:** Analysis of the pathological results of patients with single nodule localization.

	Medical glue group (*n* = 416)	Positioning hooks group (*n* = 302)
Benign lesions		
Inflammatory lesion	4	11
Atypical adenomatous hyperplasia	22	12
Hamartoma	3	2
Sclerosing hemangioma	2	1
Fibroplasia	22	9
Lymphadenopathy	8	2
Granulomatous inflammation	7	6
Alveolar epithelial hyperplasia	14	7
Malignant lesions		
Bronchial adenoma	2	1
Adenocarcinoma infiltrating	133	55
Minimally invasive adenocarcinoma	138	132
Adenocarcinoma *in situ*	60	61
Squamous cell carcinoma	1	0
Metastatic carcinoma	0	3

**Table 7:** Analysis of the pathological results of patients with multiple nodules.

	Medical glue group (*n* = 59)	Positioning hooks group (*n* = 61)
Benign lesions		
Inflammatory lesion	0	0
Atypical adenomatous hyperplasia	5	7
Hamartoma	0	0
Sclerosing hemangioma	0	0
Fibroplasia	4	4
Lymphadenopathy	0	2
Granulomatous inflammation	0	1
Alveolar epithelial hyperplasia	13	1
Malignant lesions		
Bronchial adenoma	0	0
Adenocarcinoma infiltrating	8	9
Minimally invasive adenocarcinoma	45	41
Adenocarcinoma *in situ*	23	26
Squamous cell carcinoma	0	2
Metastatic carcinoma	0	0

### 3.7 Morphological distribution of malignant tumors in the enrolled patients

**Figure 7:**
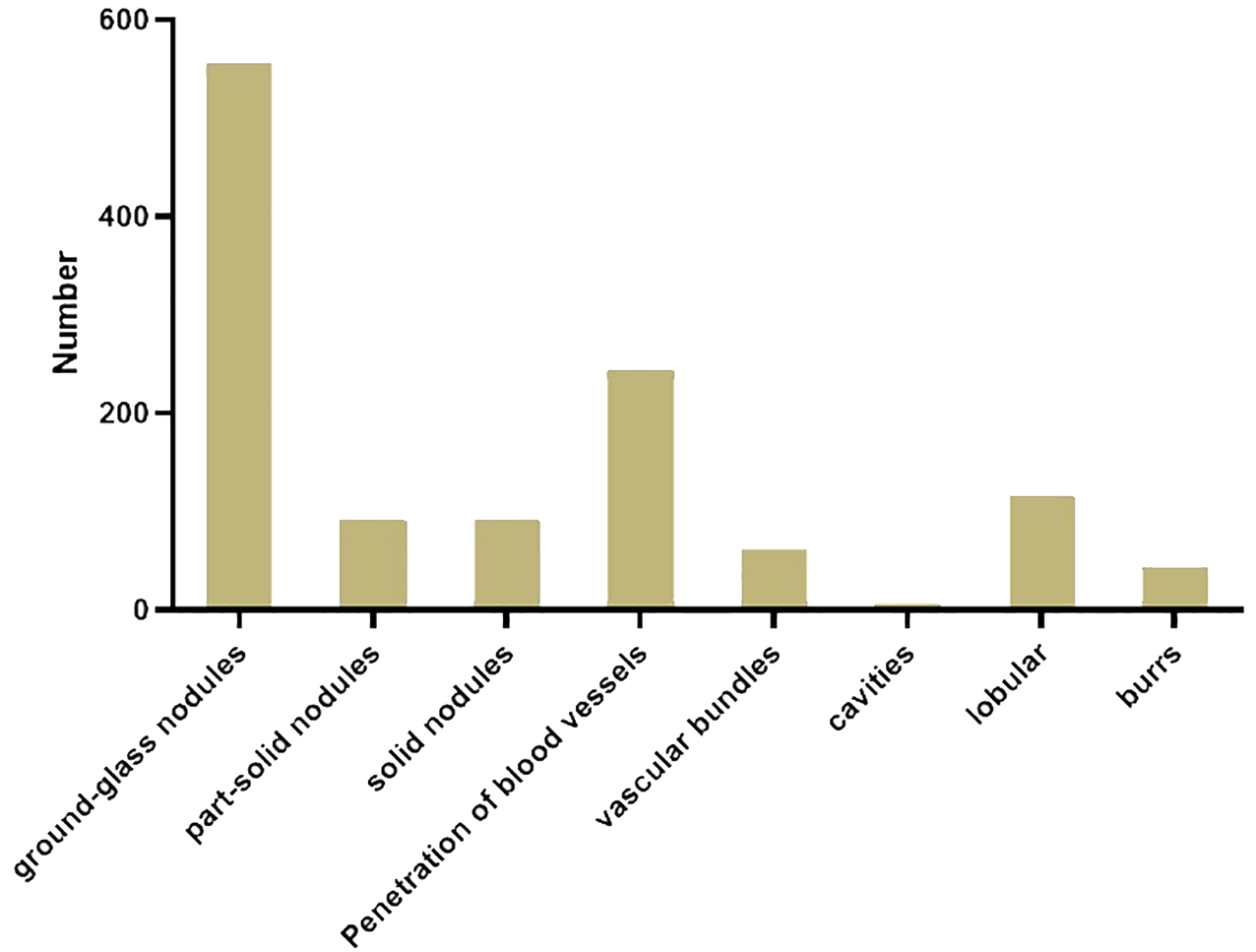
Morphological distribution of malignant tumors in the enrolled patients.

Among the patients enrolled in this study, a total of 740 nodules were diagnosed as malignant tumors. Among them, 92 nodules were solid, and 648 nodules were subsolid. The subsolid nodules included 556 ground glass opacities (no solid component) and 92 part-solid modules (both ground glass and solid components). In addition, of all the nodules diagnosed as malignant tumors, 244 had vascular penetration, 62 had vascular bundles, seven contained cavities, 117 were lobulated, and 44 had burrs (**Figure 7**).

### 3.8 Relationship between COVID-19 and lung nodule occurrence

**Figure 8:**
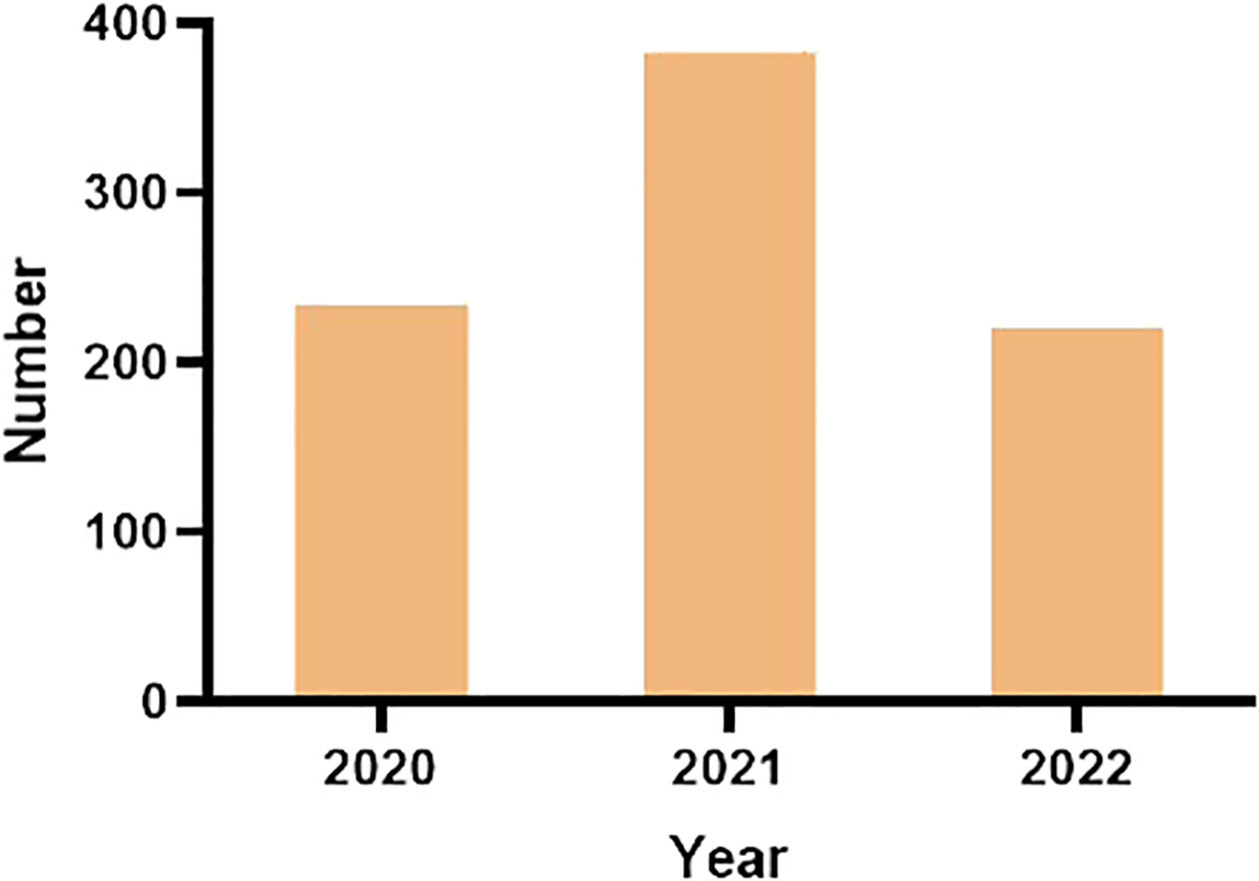
Number of preoperatively localized pulmonary nodules in 2020–2022.

The 2020–2022 period was an important period for the outbreak of COVID-19, during which the number of patients who received medical glue and localization hooks for localization increased from 233 in 2020 to 383 in 2021 and then decreased to 221 in 2022, revealing a certain positive correlation between the spread of COVID-19 and the detection rate at which patients underwent chest CT examination (**Figure 8**). In addition, it is worth noting that 2022 is only half a year.

## 4 Discussion

With the wide application of CT for the early screening of lung cancer, the mortality rate of lung cancer patients has greatly decreased (13), and the detection of GGNs has become easier. Since many of these patients have early-stage lung cancer or precancerous lesions, early surgical resection is particularly important. With the increased use of VATS in the clinic, many patients with GGN are unable to accurately localize the nodule location due to its small diameter or deep location in the lung parenchyma, resulting in prolonged operation time and exposing the patients to greater risks (14). Therefore, accurate localization of lung nodules before surgical resection is crucial (4).

Several methods for the preoperative localization of GGNs have been used in the clinic, but the ideal method has not yet been identified (15). During hookwire positioning, 2.5%–13% of cases are decoupled, leading to localization failure and increased surgical risk (16). The methylene blue dye disperses rapidly and needs to be removed as quickly as possible when applied for preoperative localization, and the dye may also cause allergic reactions (17), making the detection of GGNs challenging. Intraoperative ultrasound, while not associated with the risk of pneumothorax and hemorrhage, requires specialized equipment and has significantly reduced resolving power in patients with asthma, herpetic emphysema, diffuse emphysema, or pulmonary fibrosis due to insufficient lung tissue collapse and emphysema (18).

Among the two preoperative localization methods used in this study, medical glue, also known as tissue adhesive, is made from synthetic α-cyanoacrylate, which is nontoxic, safe, and commonly used for the preoperative localization of pulmonary nodules (19). This glue can be rapidly polymerized and cured in the presence of blood and tissue fluid anions. Numerous studies have demonstrated the safety and effectiveness of medical glues in a variety of applications, such as hemostasis, reinforcement of intestinal anastomoses or potential fluid leakage sites, wound closure, and vascular embolization (20–22). Positioning hooks are widely used in clinical practice because of their relative stability, high success rate, simplicity, and avoidance of displacement and decoupling due to respiratory movements. Our study shows that the success rate of both methods can reach 100% for the localization of a single pulmonary nodule or multiple pulmonary nodules. As the medical glue injected into the patient’s lungs can produce a hard nodule, it can be easily felt by the operator; thus, surgical resection can be accomplished. The positioning hooks are easily retrieved and dislodged from the lung tissue during surgery (23). However, in our study, the success rate of positioning hooks was also 100%, possibly because the operator could determine the location of the nodule through the hook puncture point on the lung surface and retrieve the hook, thus completing the resection of the nodule successfully.

Previous studies have shown that both medical glue and positioning hooks may cause complications (11, 12). Complications during positioning of medical adhesive may be due to local tissue damage and infection caused by positioning, detachment or migration of medical adhesive, etc. A few patients may have allergic reactions or foreign body reactions, such that the smell of medical adhesive may stimulate the patient to produce a transient cough. Complications with the positioning hook are similar to those with medical adhesive, but some patients may also experience a shallow needle entry that prevents the hook from being adequately anchored to the patient’s tissues, and if the patient is active after positioning, the hook may be dislocated, resulting in hemorrhage (16, 24). To some extent, the use of thicker positioning hooks (21G) for positioning also increases the risk of gas entry during puncture and the risk of puncturing capillaries (25), but the positioning hook (19G) used in this paper can somewhat reduce the occurrence of the above-mentioned complications. In this study, the most common complications in patients with medical glue and positioning hooks were pneumothorax and parenchymal hemorrhage. The proportions of patients with mild pneumothorax and mild hemorrhage and a single nodal location in the medical glue group were 6.97% and 8.89%, respectively. The proportions of mild pneumothorax and mild hemorrhage in patients with single nodal localization in the positioning hooks group were 8.94% and 5.63%, respectively. The incidence of pneumothorax in the positioning hooks group was greater than that in the medical glue group, but the incidence of bleeding was lower than that in the medical glue group. In addition, 1.92% of the patients in the medical glue group had medical glue covering nodules, and 3.31% of the patients in the positioning hooks group had nodules touched by the positioning hooks, which increased the difficulty of removing the nodules after resection. The proportion of patients in the medical glue group who developed medical-glue-related complications such as bleeding and glue that were indistinguishable from each other was 4.09%, and the proportion of patients who developed coughing reached 14.4%, which did not affect localization but increased the patients’ discomfort. The possible reasons for cough in patients with medical glue positioning are as follows: (1) medical glue has a certain irritating odor, which can easily cause coughing symptoms (26); (2) in the process of positioning, the injection speed is too fast or the amount of medical glue is too high; and (3) medical glue is a liquid that is more likely to diffuse to the surrounding area, especially to small bronchial tubes. However, the patients positioned by the positioning hooks did not develop significant cough. These results preliminarily indicate that positioning hooks may be more comfortable and safer than medical glue, but this conclusion requires further analysis. Among the patients in whom multiple nodes were localized, 13.56% and 21.31% of the patients in the two groups had mild pneumothorax, and 11.86% and 13.11% of the patients developed mild bleeding. These percentages were significantly greater than those of patients with single node localization, and the proportions of patients who developed overlying nodes were also greater (6.78% and 4.92%, respectively). The increased percentage of pneumothorax in both groups may be due to the multiple positioning needles in and out of the pleura used during the localization of multiple nodules. Although surgeons attempt to minimize the number of positioning needle entries and exits during positioning operations, the incidence of pneumothorax in the positioning of multiple nodes increases significantly with the number of positions, and the incidence of pneumothorax can reach 100% when the number of nodes positioned is more than five (27). The percentage of patients who experienced coughing was also greater in patients with multiple nodes localized by medical glue, reaching 18.6%. One patient had severe coughing, which resulted in the medical glue being coughed out of the trachea and needing to be repositioned. In addition, in this study, 60% of patients with single nodules and multiple nodules located by medical glue experienced postoperative pain, of whom 29 patients (6.10%) had a pain score ≥4. Most patients with positioning hooks did not have pain, and only 14 patients (3.86%) had a pain score ≥4. This may be due to the chest wall being too thick for anesthesia to reach the pleura effectively. One patient had severe hemoptysis and required emergency treatment. Postoperative bleeding may be caused by the hooking of small bronchial tubes and terminal small blood vessels during positioning of the positioning hooks. In contrast, medical glue positioning was associated with more medical-glue-related complications and a decrease in patient comfort, which is consistent with previous studies (28). Embolization, as one of the preoperative positioning complications, has been reported in very few patients, such as patients with hookwire positioning, with an incidence of 0.6% (29). In this study, air embolism was not reported in either group of patients. Therefore, considering patient acceptance and avoiding complications, it is better for patients to use positioning hooks.

To investigate whether the general characteristics of the patients could affect their lung nodule status, this study also analyzed the relationships between age, BMI, and smoking history and the size, location, and distance of the lung nodules from the pleura. The results showed that there was a correlation between patient age, BMI, and smoking status and the size of lung nodules (*p* < 0.05), but there was no significant correlation between the location of lung nodules and the distance from the pleura (*p* > 0.05). Previous studies have shown that patients’ physical conditions, such as age and BMI, are influential factors in the development of pulmonary nodules (30), which, to some extent, affects the success of preoperative localization of pulmonary nodules as well as the occurrence of complications. In addition, cigarette smokers, with cigarette classified as group I carcinogens, are prone to the development of lung nodules (31). This study also analyzed the independent risk factors for complications, but the results showed that the general clinical characteristics of the patients, lung nodules, and localization method were not factors influencing the development of complications, indicating that there was no statistically significant difference in the probability of complications between the two localization methods. Therefore, the operator’s choice of localization method for lung nodules should be tailored to the individual.

In the United States, 30% of the 1.6 million patients who undergo CT examination each year have pulmonary nodules (32). Approximately 5% of the nodules were diagnosed as malignant tumors. Nodules can be divided into solid nodules and subsolid nodules. Subsolid nodules included ground glass nodules and partial solid nodules. Our study analyzed the benign, malignant, and morphological characteristics of 957 nodules in all patients. Among them, 740 nodules (77.3%) were diagnosed as malignant tumors, including 92 solid nodules, 92 partial solid nodules, and 556 ground glass nodules. The greater proportion of malignant tumor nodules may be because our study included only patients from one hospital, and the time span was shorter. In addition, 244 nodules had vascular perforations, 62 nodules had vascular bundles, seven nodules had vacuoles, 117 nodules were lobulated, and 44 nodules were spiculated. Studies have shown that there is a certain relationship between the above-mentioned morphological characteristics and the increased risk of malignant tumors and invasiveness (33).

The years 2020–2022 coincided with the outbreak of COVID-19, and 2021 was the most severe year. The number of patients who underwent chest CT and VATS increased significantly during this period (34). The statistics of this study regarding the number of lung nodules in 2020–2022 also demonstrated a positive correlation between the detection of lung nodules and the spread of COVID-19.

There are two obvious drawbacks to this study. The first is the lack of randomization in this study, and the second is that this study was a retrospective analysis conducted at a single site, which may have had some impact on the results of the comparison of the safety and efficacy of the two types of preoperative positioning, the medical glue and the positioning hooks. Despite these limitations, our study provides new insights into preoperative positioning techniques. In summary, medical glue itself has advantages, namely: (1) as a tissue adhesive, the positioning operation is simple and can avoid the decoupling phenomenon due to the patient’s respiratory movement or movement and (2) it can quickly stop bleeding, and the adhesion process is painless, which reduces the need to restrict the patient’s activities, and the operation time is relatively flexible. However, this study revealed that the use of medical glue easily causes patients to cough. Although coughing did not cause positioning failure or complications, there is still a certain safety risk. In addition, the use of medical glue is more demanding, the speed of injection is greater, and the use of medical glue is also prone to nodular coverage, bleeding, and glue indistinguishability compared with positioning hooks, which are more likely to have complications. The positioning of the hooks is simple, and there is basically no cough. In addition, positioning the material of the hooks is difficult, and the hooks are more tactile during surgery. However, the operator needs to avoid large blood vessels. There are cases of dislocation and displacement of the positioning hooks, which require a certain depth of entry into the pleura, and the patient needs respiratory cooperation. Based on the above-mentioned details, both localization techniques can effectively achieve preoperative localization of pulmonary nodules. Therefore, the selection of an appropriate localization method for patients should be based on factors such as economic costs, infrastructure, the experience of the attending physician, and other relevant considerations.

## Author contributions

HW: Data curation, Investigation, Writing – original draft, Writing – review & editing. JS: Conceptualization, Writing – review & editing. RF: Writing - review & editing, Investigation. HL: Formal analysis, Resources, Writing – original draft, Writing – review & editing. TH: Resources, Data curation, Writing – original draft, Writing – review & editing. DL: Resources, Writing - original draft. CC: Writing – original draft,Formal analysis. PZ: Formal analysis, Writing – original draft, Writing – review & editing. MD: Writing – original draft. DC: Data curation, Methodology, Writing – original draft.
